# Monocyte‐predominant engraftment, cytokine levels and early transplant‐related complications in pediatric hematopoietic stem cell recipients

**DOI:** 10.1002/cam4.1912

**Published:** 2019-01-28

**Authors:** Natalia Maximova, Marilena Granzotto, Francesca Barbieri, Annalisa Marcuzzi, Alberto Tommasini, Lorenzo Monasta, Roberto Simeone, Davide Zanon, Roberto Sala

**Affiliations:** ^1^ Institute for Maternal and Child Health—IRCCS “Burlo Garofolo” Trieste Italy; ^2^ Clinical Epidemiology and Public Health Research Unit Institute for Maternal and Child Health—IRCCS “Burlo Garofolo” Trieste Italy; ^3^ Department of Laboratory Medicine ASUITS Trieste Italy; ^4^ Department of Transfusion Medicine ASUITS Trieste Italy; ^5^ Department of Medicine, Surgery and Health Sciences University of Trieste Trieste Italy; ^6^ Department of Medicine and Surgery University of Parma Parma Italy

**Keywords:** cytokines, defibrotide, hematopoietic stem cell transplantation, monocyte‐predominant engraftment, pediatric, short‐term transplant‐related complication

## Abstract

Myeloablative conditioning is a well‐established procedure that precedes hematopoietic stem cell transplantation (HSCT), particularly in pediatric patients. In the period directly following transplantation, several factors may contribute to complications that lead to the activation or damage of endothelial cells, involved in the pathogenesis of vascular endothelial syndromes (VES). However, to date, sufficiently specific and sensitive diagnostic markers for the various forms of VES have not been identified. This was a retrospective single‐center study of patients who underwent allogeneic HSCT. For this cohort of patients, parameters including type of engraftment, donor characteristics, and cytokine production were measured and correlated with a high prevalence of short‐term complications after HSCT. The aim of this study was to identify specific parameters useful for improving diagnostics and predicting adverse effects in VES. We confirmed that monocyte‐predominant engraftment was related to a higher risk for an early transplant‐related complication termed sinusoidal obstruction syndrome (SOS). The increased production of specific cytokines, in particular RANTES, represents a marker associated with prevalent engraftment. In addition, patients undergoing prophylaxis with defibrotide had “classical” engraftment, a common cytokine profile and a lower incidence of life‐threatening transplant‐related complications. The beneficial effect of defibrotide might be a starting point for developing selective prophylaxis for patients with monocyte engraftment to prevent severe early transplant‐related complications.

## Abstract 2

The onset of vascular endothelial syndromes after hematopoietic stem cell transplantation is significantly associated with monocyte‐predominant engraftment (*P* < 0.0001). The increased production of specific cytokines, in particular RANTES, represents a marker associated with monocyte‐predominant engraftment. There is close association between the prophylactic administration of defibrotide, a powerful endothelial protector, and the incidence of vascular endothelial syndromes (*P* < 0.0001). Given the high cost of defibrotide, selective prophylaxis may be considered for patients with extremely high values of RANTES or with monocyte engraftment.

## 1 INTRODUCTION

Hematopoietic stem cell transplantation (HSCT) is commonly used to treat pediatric disease, especially hematological and oncological disorders or congenital errors. Particularly aggressive chemotherapy treatments are part of most transplantation conditioning regimens. Myeloablative conditioning refers to the administration of a maximum tolerated dose of chemotherapy or radiotherapy to destroy functional hematopoiesis and host pathological cells; thus, eradicating all immunocompetent cells and creating space in the bone marrow microenvironment to allow new cells to engraft and prevent rejection. Within a month after transplantation, patients may develop complications caused by the direct or indirect toxicity of chemotherapy or radiotherapy, which leads to endothelial dysfunction. Examples are the production of cytokines by damaged tissues and the translocation of bacteriological endotoxins from the affected gastrointestinal tract. During HSCT, endothelial cells (ECs) are activated by treatments including granulocyte‐colony stimulating factor (G‐CSF) or calcineurin inhibitors.^1^

These elements, together with the engraftment of donor cells, which can be complicated by allogeneic reactions, leads to EC damage.^2^

Substantial scientific evidence suggests that some post‐HSCT early complications, such as sinusoidal obstruction syndrome (SOS),^3^ capillary leak syndrome,^4^ engraftment syndrome,^5^ transplant‐associated thrombotic microangiopathies (TMA),^6^ and graft‐vs‐host disease (GVHD),^7^, ^8^ originate from localized or systemic EC damage.

Several recent studies suggested different combinations of cytokines and chemokines might be specific markers for diagnosis and used to distinguish between specific forms of vascular endothelial syndrome (VES).^2^, ^9^, ^10^, ^11^, ^12^ However, other studies have presented contrasting data, and to date no specific or sufficiently sensitive marker has been identified for the different forms of VES.

Typical engraftment is commonly defined as a rapid repopulation of polymorphic nucleated cells (PMN) and monocytes^13^ followed by a generally slow lymphocyte recovery. A small number of studies have reported a non‐typical hematopoietic engraftment, for example, predominantly monocyte engraftment^14^. Over the past 3 years, our institute has seen a considerable increase in non‐classical engraftment, with a clear monocyte predominance compared with other cell lineages from the very early stages of the engraftment.

Therefore, our retrospective study investigated the relationship between predominantly monocyte repopulation and the appearance of early complications including VES and GVHD, and the potential prognostic significance of such complications.

## 2 MATERIALS AND METHODS

### 2.1 Patients

A retrospective single‐center study was carried out at the Institute for Maternal and Child Health—IRCCS “Burlo Garofolo”, Pediatric Transplant Centre in Trieste, Italy. The study protocol was approved by the Ethics Committee of the IRCCS “Burlo Garofolo” (reference no. 1105/2015). Patients were asked to authorize the release of information for research purposes. The medical records of all patients, who underwent allogeneic HSCT at our Center between January 2010 and December 2017 were analyzed. The medical records of patients aged >18 years who were further treated with subsequent transplantation, were not considered. Moreover, subjects for whom post‐transplantation samples were not available were also excluded from the study.

Inclusion criteria were: age of recipient <18 years at the time of transplantation, patients treated with their first allogeneic transplantation, myeloablative conditioning regimen, post‐transplantation follow‐up of at least a month after transplantation, and documented engraftment.

### 2.2 HSCT procedure

Allogeneic transplantations were performed after myeloablative conditioning based on Total Body Irradiation (TBI) at a dose of 12 cGy, divided into six sessions for patients aged >2 years and suffering from acute lymphoblastic leukemia. All other patients received busulfan orally (360 mg/m^2^, dosage subject to adjustment according to therapeutic drug monitoring) or treosulfan (12‐14 g/m^2^ based on recipient's age). Patients undergoing a matched unrelated donor (MUD), haploidentical or sibling transplantation—in the case of haemoglobinopathy—also received antilymphocyte serum. Anti‐rejection prophylaxis included tacrolimus for sibling transplantations, tacrolimus plus mycophenolate mofetil (MMF) for MUD transplantations, and addition of cyclophosphamide for haploidentical transplantations.

### 2.3 Type of engraftment

A hemocytometer count was performed for each patient using a DXH800 Beckman Coulter haemocytometer (Beckman Coulter S.r.l., Cassina de’ Pecchi [MI], Italy) to assess the complete blood count and white blood cell populations. At the same time, blood samples of patients were also evaluated using a cytofluorometric technique to confirm the hemocytometer count related to the presence of different populations of leukocytes and to study the distribution of lymphocytes. Anti‐CD45 antibodies were used for leukocyte and lymphocyte populations, anti‐CD19 antibodies for the identification of B lymphocytes, anti‐CD4 and anti‐CD8 antibodies for lymphocytes, and anti‐CD16/56 to identify NK cells. Blood samples were analyzed using a flow cytometer (Navios; Beckman Coulter). For each analysis, 30 000 events were acquired. Data were subsequently analyzed using a gating strategy to identify cells of interest.

We considered “predominant‐monocytes” engraftment when the percentage of monocytes was higher than any other leukocyte lineage in the first stage of engraftment, beginning when the total leukocytes exceeded 100 cells/mmc. The preponderance of monocytes had to be documented in at least two different determinations performed at an interval of 2 or 3 days. Engraftment was defined as “classical” in all other cases, ie when the count of lymphocytes and/or PMN was higher than that of monocytes.

For flow cytometric analysis, monocytes were gated according to their forward and side scatter profile and further characterized due to their CD14 and CD16 surface expression. Monocyte subpopulations CD14++CD16− was classified “classical” (M1), CD14++CD16+ “intermediate” (M2) and CD14+CD16++“non classical” (M3).

To obtain a more accurate statistical profile, we standardized engraftment time from various stem cell sources: in the case of PBSCs, the engraftment timeline was shortened by 4 days because the engraftment time of PBSCs is approximately 4 days shorter than that of bone marrow stem cells.^15^

In contrast to previous studies^16^ and based on our experience, the engraftment of umbilical cord stem cells was standardized by shortening the timeline to 4 days instead of 5. Chimerism analyses were performed using a semiquantitative polymerase chain reaction (PCR) approach based on the amplification of short tandem repeats (STR).

### 2.4 Prophylaxis with defibrotide

Between January 2010 and June 2014, all patients receiving an allogeneic transplantation underwent SOS prophylaxis with defibrotide. From July 2014 until September 2015, only patients at high risk of developing SOS underwent prophylaxis with defibrotide. Since September 2015, the administration of defibrotide in our center has been restricted to treating SOS or particularly severe VES. Defibrotide prophylaxis started on the first day and lasted until 28 days after conditioning, at a dose of 25 mg/kg divided into four administrations per day.

### 2.5 Early complications related to transplantation

Sinusoidal obstruction syndrome was diagnosed based on the guidelines reported by Corbaciouglu and approved by the European Group for Blood and Marrow Transplantation (EBMT Group).^17^ GVHD was diagnosed, and its relevant severity level assigned, based on classical criteria.^18^ Diagnosis of capillary leak syndrome was formulated based on criteria published by Nürnberger^4^ in 1997. Engraftment syndrome was diagnosed following Spitzer's definition.^5^, ^19^ Diagnosis of idiopathic pneumonia syndrome was formulated based on Clark's criteria.^20^ Diagnosis of transplantation‐associated TMA was defined following the diagnostic contents in Blood and Marrow Transplant Clinical Trials Network (BMT CTN) Toxicity Committee Consensus Definition for TMA.^20^

### 2.6 Analysis of cytokines and chemokines

The analysis of 27 cytokines and chemokines, namely IL‐1β, IL‐1ra, IL‐2, IL‐4, IL‐5, IL‐6, IL‐7, IL‐8, IL‐9, IL‐10, IL‐12(p70), IL‐13, IL‐15, IL‐17, Eotaxin, FGF basic, G‐CSF, GM‐CSF, IFN‐γ, IP‐10, MCP‐1 (MCAF), MIP‐1α, PDGF‐bb, MIP‐1β, RANTES (CCL5), TNF‐α, and VEGF was carried out on plasma samples with multiple immunoassays, using a bead‐based magnetic sensor (27 human‐Bio‐Plex assay; BIO‐RAD Laboratories, Milan, Italy) following the manufacturer's instructions. Data were acquired by a Bio‐Plex 200 reader, and a digital processor and BIO‐RAD, Hercules, California, USA software converted data into median fluorescence intensity and concentration (pg/μL).

Cytokines and chemokines were measured in 26 patients. For 20 subjects receiving myeloablative chemotherapy, samples were obtained before conditioning, while for six subjects treated with chemoradiotherapy condition, baseline samples were obtained after TBI.

A second determination was carried out on 26 samples from the same patients, collected 2‐3 days after engraftment. The last determination was performed on 18 samples from patients who developed an early complication associated with transplant procedure, including VES or grade III‐IV GVHD. Samples were collected 1 or 2 days before the appearance of clinical signs of complication.

The adopted methodology allowed ULOQ (Upper Limit of Quantification) and LLOQ (Lower Limit of Quantification) cytokine values to be assigned a numeric value higher than the determination threshold value. The highest determinable threshold value for RANTES was 203 356.8 pg/μL. Any determination having a ULOQ result was therefore assigned a value of 205 000.0 pg/μL. For IL‐2, the lowest determinable value was 0.9 pg/μL. Any determination with an LLOQ was assigned a value of 0 pg/μL.

### 2.7 Statistics

Collected data were analyzed using descriptive statistics to determine the distribution and frequency of the variables. Continuous variables were expressed as the mean and standard deviation (SD), while categorical variables were expressed as the frequency, absolute or percentage value. Student's *t* tests were used to compare different groups of patients. Two‐tailed Fisher exact test was performed to assess the association between categorical variables. Paired Student's *t* tests were used to compare pre‐ and post‐transplant paired data in the same group of patients. *P*‐values <0.05 were considered as statistically significant. To avoid problems of separability, we carried out simultaneous analyses adopting Firth's penalized likelihood approach to logistic regressions, considering the type of engraftment as the main dichotomous outcome. Statistical analyses were performed using WinStat (v.2012.1; In der Breite 30, 79189 Bad Krozingen, Germany), Prism 5 for Windows (7825 Fay Avenue, Suite 230, La Jolla, CA 92037 USA. Software, Inc.) and Stata/IC 14.2 (StataCorp LLC, College Station, TX).

## 3 RESULTS

We examined the medical records of 87 patients who underwent allogeneic transplant at our Institute from January 2010 until December 2017. Eight patients were excluded from the study for the following reasons: four patients were ≥18 years of age at the time of transplant; three patients had a conditioning regime that was not myeloablative; and insufficient biological samples were available for one patient, who died during the first month after transplant. The remaining 79 patients constituted our study group with a prevalence of males vs females (62% and 38%, respectively) and with a mean age of 8.7 years at the time of transplant.

The indication for transplantation in almost half of the cases was high‐risk acute lymphatic leukemia (46%), followed by myelodysplastic syndrome and acute myeloid leukemia (19% and 18%, respectively).The conditioning regimen was myeloablative in all cases, which were subdivided between total body irradiation (TBI) and myeloablative chemotherapy (MCHT) groups, with a slight prevalence toward MCHT (57% vs 43%). The demographic data of the study group are shown in Table 1.

**Table 1:** Patient demographics

Pretransplant baseline characteristics	Whole cohort
Number of patients (%)	79 (100)
Sex	
Male (%)	49 (62)
Female (%)	30 (38)
Age at transplant, years, mean (±SD)	8.7 (4.9)
Underlying disease, number (%)	
Acute lymphoblastic leukemia	36 (46)
Acute myeloid leukemia	14 (18)
Myelodysplastic syndrome	15 (19)
Inborn error	7 (9)
Hemoglobinopathy	3 (4)
Solid tumor	4 (5)
Disease stage, number (%)^a^	
Early	24 (30)
Intermediate	26 (33)
Late	15 (19)
Myeloablative conditioning, number (%)	
MCHT‐based	45 (57)
TBI‐based	34 (43)
Donor type, number (%)	
Matched related donor	28 (35)
Matched unrelated donor	44 (56)
Haploidentical donor	7 (9)
Graft source, number (%)	
Bone marrow	63 (80)
Peripheral blood stem cells	13 (16)
Umbilical cord blood	3 (5)

MCHT, myeloablative chemotherapy; TBI, total body irradiation; SD, standard deviation.

This classification is applied to patients with acute leukemia and myelodysplastic syndrome only.^33^

a Disease stage was defined according to previously published classification.

### 3.1 Factors that influence engraftment

Based on the main objective of the study, we divided our population into two groups, differentiating them by the type of patient engraftment. The first group comprised 53 patients (67%) with a “classic” engraftment (“classic engraftment group”), and the other group comprised 26 patients (33%) with a clear monocytic prevalence in the first phase of the engraftment (“monocyte‐predominant engraftment group”). We analyzed the immunophenotype expressed by these monocytes and found that all samples had an extremely homogeneous monocyte population composed exclusively of monocytes with a classical immunophenotype (M1).

Donor chimerism was analyzed in a time frame from day +20 to day +30 in all patients. Among 26 patients with monocyte engraftment, full donor chimerism was documented in 23 patients. The donor chimerism was almost complete in the three remaining patients: one had 99.2% donor chimerism, and two had 98.8% and 98.9% donor chimerism, respectively.

The factors related to the bone marrow niche, infused stem cells, conditions of the recipients and possible concomitant treatments in both groups are shown in Table 2. There was no statistically significant difference in the accumulation of iron in bone and osteoporosis in the two groups. Factors significantly associated with “classical engraftment” were the use of bone marrow as a source of stem cells; use of a matched related donor; age of the donor below 18 years; G‐CSF stimulation; and use of myelotoxic drugs. In contrast, factors associated with “monocyte predominant engraftment” were the use of PBMC as a source of stem cells and age of the donor above 18 years.

**Table 2:** Factors affecting engraftment during allogeneic HSCT

Variables	“Classical” engraftment	Monocyte predominant engraftment	*P*‐value
Type of engraftment, number	53	26	—
Haematopoietic niche‐related factors, number (%)			
Bone iron overload^a^	28 (53)	16 (62)	0.483
Osteoporosis^b^	12 (23)	7 (27)	0.781
Graft‐related factors			
Graft source, number (%)			
Bone marrow	47 (89)	16 (62)	0.008
PBSC	4 (8)	9 (35)	0.007
Cord blood	2 (4)	1 (4)	1.000
Donor type, number (%)			
Matched related donor	23 (43)	5 (19)	0.046
Matched unrelated donor	27 (51)	17 (65)	0.241
Haploidentical donor	3 (6)	4 (15.)	0.210
Donor age, number (%)			
<18 y	23 (43)	3 (12)	0.005
≥18 y	30 (57)	23 (88)	
Number of TNC × 10^8^/kg infused, mean (SD)^c^	5.7 (2.5)	6.5 (2.4)	NS
Number of CD34 × 10^6^/kg infused, mean (SD)^d^	10.5 (5.4)	9.5 (4.1)	NS
Patient‐related engraftment‐concomitant factors, number (%)			
G‐CSF stimulation	15 (28)	1 (4)	<0.001
Virus infection	15 (28)	8 (31)	1.000
Myelotoxic drugs	12 (23)	6 (23)	1.000
Defibrotide prophylaxis, number (%)	42 (79)	2 (8)	<0.001

G‐SCF, granulocyte colony‐stimulating factor; HSCT, hematopoietic stem cell transplantation; NS, not significant; PBCS, peripheral blood stem cells; SD, standard deviation; TNC, total nuclear cells.

a Magnetic resonance imaging was used to measure iron concentrations in the liver, spleen, pancreas and bone.^34^

b Dual energy X‐ray absorptiometry bone densitometry was used.

c Applied to bone marrow only.

d Applied to PBCS only.

Multivariate analysis (Table 3) showed that a subject receiving the transplant from a donor ≥18 years of age had a much higher risk of monocyte engraftment if compared with a subject receiving cells from a donor <18 years. Moreover, cord blood transplantation significantly increased the probability of monocyte predominant engraftment if compared with bone marrow. Finally, of note, the use of defibrotide prophylaxis was associated with a much lower risk of monocyte predominant engraftment.

**Table 3:** Multivariate logistic regression analysis on factors associated with monocyte predominant engraftment vs “classical” engraftment, during allogeneic HSCT

Variables	Odds ratio	95% confidence interval	*P*‐value
Bone iron overload (1. Yes vs 0. No)	3.000	0.421‐21.385	0.273
Osteoporosis (1. Yes vs 0. No)	1.257	0.143‐11.010	0.836
Graft source (bone marrow as reference)			
PBSC	8.279	0.456‐150.201	0.153
Cord blood	134.952	1.489‐12232.43	0.033
Donor type (matched related donor as reference)			
Matched unrelated donor	0.041	0.001‐2.270	0.119
Haploidentical donor	0.417	0.010‐17.616	0.647
Donor age (1. ≥18 y vs 0. <18 y of age)	148.688	1.625‐13607.13	0.030
G‐CSF stimulation (1. Yes vs 0. No)	0.091	0.003‐3.079	0.182
Virus infection (1. Yes vs 0. No)	0.737	0.096‐5.643	0.769
Myelotoxic drugs (1. Yes vs 0. No)	0.533	0.051‐5.559	0.599
Defibrotide prophylaxis (1. No vs 0. Yes)	134.113	4.273‐4209.074	0.005

G‐CSF, Granulocyte‐colony stimulating factor; HSCT, hematopoietic stem cell transplantation; PBSC, peripheral blood stem cells.

### 3.2 Differences in leukocyte engraftment and early transplant‐related complications

The results of this analysis are shown in Table 4. The time at which the engraftment of total leukocytes and polymorphonuclear cells occurred was similar in both groups. As expected, the percentage of monocytes at engraftment was significantly higher in the “monocyte predominant engraftment” group. Regarding transplant‐related complications, subjects with “monocyte predominant engraftment” had a higher mean grade of mucositis than subjects with classical engraftment. While infections from virus or bacteria occurred at similar rates in the two groups, fungal infections were recorded only in subjects with classical engraftment.

**Table 4:** Transplant‐related features: differences between two groups during the engraftment phase

Variables	“Classical” engraftment (n = 53)	Monocyte predominant engraftment (n = 26)	*P*‐value
Time of WBC engraftment (≥1000/mm^3^), days; mean (±SD)	22 (9.9)	19.4 (5.8)	0.271
Time of neutrophil engraftment, days; mean (±SD)			
ANC ≥ 500/mm^3^	20 (8.2)	19 (5.7)	0.650
ANC ≥ 1000/mm^3^	26 (11.0)	26 (10.3)	0.849
Time of monocyte engraftment, days; mean (±SD)			
Monocyte ≥ 500/mm^3^	23 (6.4)	18 (6.3)	0.053
Monocyte ≥ 1000/mm^3^	34 (7.1)	35 (8.7)	0.742
Maximum percentage of neutrophils at engraftment, mean (±SD)	98 (18.9)	43 (10.7)	<0.001
Maximum percentage of monocyte at engraftment, mean (±SD)	26 (13.2)	59 (11.3)	<0.001
WHO grade mucositis, mean (±SD)	2 (0.8)	3 (1.0)	<0.001
Infectious complications, number (%)	37 (70)	13 (50)	0.627
Bacterial infection	9 (17)	6 (23)	0.551
Fungal infection	11 (21)	0 (0.0)	0.013
Virus infection	17 (32)	7 (37)	1.000
GVHD, number (%)			
None	34 (64)	15 (58)	0.627
I/II overall grade	13 (25)	3 (12)	0.239
III/IV overall grade	6 (11)	8 (31)	0.057
Vascular endothelial syndromes, number (%)	4 (8)	23 (88)	<0.001
Sinusoidal obstruction syndrome	2 (4)	6 (23)	0.014
Engraftment syndrome	1 (2)	3 (12)	0.102
Capillary leak syndrome	1 (2)	6 (23)	0.004
Transplant‐associated thrombotic microangiopathy	0	5 (19)	0.003
Idiopathic pneumonia syndrome	0	3 (12)	0.033
Diffuse alveolar hemorrhage	0	0	—

ANC, absolute neutrophil count; GVHD, Graft vs host disease; SD, standard deviation; WBC, white blood cell; WHO, World Health Organization.

From our data analysis, no significant associations emerged between the type of engraftment and the degree of GVHD. In contrast, the onset of VES was significantly associated with monocyte engraftment (88% vs 8%, *P* < 0.001). Considering the specific clinical forms constituting VES, we found a significant difference for all clinical forms engraftment syndrome.

### 3.3 Cytokine profile at baseline and engraftment phase and the proximity of complications

Analyzing the differences between baseline cytokine levels in the two groups of patients, the only statistically significant difference was detected for IL‐1ra levels, which was higher in the monocyte engraftment group. Of note, RANTES levels were very high in the groups of patients at baseline and there was a clear trend toward higher levels in the monocyte predominant group (97 128.2 ± 97 878.9 pg/mL in the “classic” group vs 12 5936.1 ± 92 615.2 pg/mL in the monocytosis group, normal values up to 25 450 pg/mL). It is likely that this difference did not reach statistical significance only because several samples in the monocyte prevalent group had RANTES levels at the Upper Limit of Quantification (ULOQ). For statistical calculations, the values of these samples were set at the upper limit of the range, ie 220 000 pg/mL. ULOQ samples were significantly more common in the monocyte group compared with the “classic” group (19/26 vs 3/26, *P* < 0.05). Overall, these data suggest that higher RANTES levels at baseline are associated with monocyte predominant engraftment. Patients expressing normal RANTES value had MDS, inborn errors or congenital immunodeficiency and, therefore, had never undergone chemo‐ or radiotherapy.

At the time of engraftment, significant differences in IL‐1ra, IL‐4, IL‐5, PBGF‐BB and TNF‐α values were detected between the two groups (*P* < 0.05) with higher levels in the monocyte predominant group (Table 5). RANTES levels decreased to near normal values without significant differences between the two groups.

**Table 5:** Baseline and post‐engraftment cytokine and chemokine profile of HSCT recipients

Cytokine/chemokine (pg/mL), mean (±SD)	“Classical” engraftment		Monocyte predominant engraftment		*P*‐value	
	Baseline	Engraftment	Baseline	Engraftment	Baseline^a^	Engraftment^b^
IL‐1β	2.8 (1.0)	2.1 (0.9)	4.5 (3.9)	2.8 (1.1)	NS	NS
IL‐1ra	193.1 (91.7)	137.4 (91.6)	443.7 (501.0)	247.7 (172.5)	0.049	0.049
IL‐2	15.6 (7.2)	9.6 (2.7)	15.0 (7.3)	12.4 (8.9)	NS	NS
IL‐4	3.1 (1.5)	1.3 (1.1)	3.9 (2.1)	3.0 (1.8)	NS	0.011
IL‐5	1.7 (2.0)	0.8 (1.4)	3.4 (3.1)	2.9 (2.0)	NS	0.010
IL‐6	73.0 (167.9)	22.5 (23.4)	73.1 (174.1)	23.7 (29.7)	NS	NS
IL‐7	5.5 (3.8)	3.9 (4.1)	7.2 (4.7)	7.0 (2.8)	NS	NS
IL‐8	27.4 (32.4)	29.0 (11.9)	95.0 (220.5)	28.9 (26.0)	NS	NS
IL‐9	153.0 (68.7)	72.5 (33.0)	261.0 (406.1)	245.7 (706.2)	NS	NS
IL‐10	9.3 (8.1)	5.5 (2.0)	7.6 (3.5)	5.7 (1.9)	NS	NS
IL‐12 (p70)	19.2 (9.6)	9.7 (4.6)	22.0 (15.7)	14.0 (10.1)	NS	NS
IL‐13	4.4 (2.0)	4.2 (4.9)	7.1 (6.3)	4.6 (2.4)	NS	NS
IL‐15	14.7 (4.5)	11.8 (6.3)	13.7 (6.6)	19.2 (13.5)	NS	NS
IL‐17	50.4 (18.8)	30.8 (8.4)	63.0 (34.7)	37.4 (14.0)	NS	NS
Eotaxin	89.4 (44.3)	98.2 (47.2)	85.3 (33.4)	99.1 (40.3)	NS	NS
Basic FGF	37.5 (14.9)	23.6 (6.5)	39.7 (17.0)	27.3 (9.6)	NS	NS
G‐CSF	152.6 (211.8)	119.4 (123.7)	148.7 (87.6)	600.3 (2210.5)	NS	NS
GM‐CSF	101.2 (35.1)	89.9 (45.1)	92.3 (54.2)	71.1 (43.6)	NS	NS
IFN‐ γ	57.6 (35.7)	35.3 (25.5)	76.4 (45.1)	53.4 (28.2)	NS	NS
IP‐10	583.7 (763.7)	646.1 (433.8)	449.8 (563.7)	314.2 (134.3)	NS	NS
MCP‐1	25.2 (34.8)	14.5 (8.6)	17.3 (11.0)	18.9 (10.2)	NS	NS
MIP‐1α	6.9 (2.2)	6.2 (4.0)	16.1 (27.8)	6.7 (3.1)	NS	NS
PDGF‐BB	1136.9 (1271.7)	107.8 (8972)	890.1 (994.4)	267.0 (192.5)	NS	0.011
MIP—1b	126.8 (108.5)	77.8 (37.5)	155.3 (191.6)	69.1 (38.5)	NS	NS
RANTES	97128.2 (97878.9)	2188.8 (2333.0)	125936.1 (92615.2)	19269.8 (38232.1)	NS	NS
TNF‐α	55.6 (23.7)	33.8 (12.29)	70.7 (32.9)	60.2 (40.1)	NS	0.017
VEGF	39.8 (39.0)	20.1 (84.3)	35.6 (25.4)	18.9 (7.8)	NS	NS

HSCT, hematopoietic stem cell transplantation; IL, interleukin; NS, not significant; SD, standard deviation.

a *P*‐value for the comparison of baseline values between Classical vs Monocyte predominant engraftment

b *P*‐value for the comparison of engraftment values between Classical vs Monocyte predominant engraftment.

Finally, we analyzed changes in cytokine expression immediately before the appearance of clinical signs of early post‐transplant complications attributable to endothelial activation (GVHD or VES). We included 10 patients with SOS, six patients with GVHD III‐IV degree and two patients with ES. For statistical reasons, given the small number of samples available for each type of complication, we examined the cytokine profile in all 18 patients taken together, making a comparison between the baseline levels and the levels found before the onset of symptoms. Statistically significant differences were documented for IL‐1ra, IL‐2, IL‐5, IL‐7, IL‐9, IL‐15, Eotaxin, Basic FGF, GM‐CSF, MCP‐1 and RANTES (Table 6). IL‐2 levels in the analyzed samples were particularly interesting. Of the 18 samples collected immediately before the development of complications, the IL‐2 values were below the LLOQ in 15 patients (83.4%) and, therefore, were not determinable. Furthermore, samples with IL‐22 below the LLOQ were found in eight of 10 SOS patients, five of six GVHD grade III‐IV patients, and two ES patients. The baseline IL‐2 values were within normal limits for all patients.

**Table 6:** Cytokines and chemokine profile in the short‐term complications of HSCT evaluated a few days before onset

Cytokine/chemokine (pg/mL), mean (±SD)	Sinusoidal obstruction syndrome (n = 10)		Graft vs host disease III‐IV grade (n = 6)		Engraftment syndrome (n = 2)		*P*‐value^a^
	Baseline	Pre‐onset	Baseline	Pre‐onset	Baseline	Pre‐onset	
IL‐1β	6.5 (5.0)	3.8 (1.8)	2.9 (1.7)	3.0 (17)	2.6 (0)	3.1 (0.1)	NS
IL‐1ra	587.6 (633.2)	132.5 (66.2)	342.3 (241.8)	135.4 (92.7)	151.3 (12.1)	83.5 (3.0)	0.013
IL‐2	18.2 (7.2)	1.0 (2.2)	11.4 (9.2)	1.2 (2.9)	10.6 (0.5)	0	<0.001
IL‐4	5.3 (1.9)	3.9 (1.3)	3.3 (1.9)	4.4 (1.8)	3.1 (0)	5.1 (0.8)	NS
IL‐5	9.5 (3.3)	12.9 (5.9)	2.2 (29.9)	48.7(40.5)	2.1 (0.6)	15.1 (1.7)	0.006
IL‐6	128.9 (230.9)	31.9 (14.3)	12.3 (62.2)	78.6 (63.5)	10 (0.3)	27.2 (1.3)	NS
IL‐7	5.4 (7.9)	34.1 (5.4)	5.8 (7.7)	21.0 (4.3)	5.8 (1.0)	28.9 (4.4)	<0.001
IL‐8	162.6 (294.0)	42.4 (13.4)	21.1 (71.1)	80.8 (59.9)	14.5 (0.7)	44.6 (4.3)	NS
IL‐9	206.5 (124.0)	50.0 (20.0)	121.4 (77.0)	73.6 (43.4)	73.7 (22.8)	47.9 (2.1)	0.002
IL‐10	9.3 (3.4)	9.6 (9.1)	5.3 (7.1)	14.9 (10.7)	4.6 (0.3)	7.3 (0.3)	NS
IL‐12 (p70)	27.4 (16.1)	23.9 (20.4)	15.0 (11.7)	16.4 (6.3)	8.6 (0.7)	10.8 (0.5)	NS
IL‐13	10.6 (7.8)	11.9 (6.8)	5.0 (6.3)	10.7 (5.7)	3.9 (0.4)	13.6 (3.0)	NS
IL‐15	15.9 (6.0)	53.8 (23.5)	10.8 (24.5)	30.0 (43.7)	7.5 (1.1)	89.3 (0.6)	<0.001
IL‐17	76.2 (34.2)	39.3 (24.8)	44.9 (37.7)	58.8 (55.0)	35.6 (6.5)	32.9 (4.6)	NS
Eotaxin	94.4 (25.4)	166.3 (35)	87.4 (52.5)	113.9 (85.8)	61.8 (8.3)	75.6 (3.1)	0.006
Basic FGF	48.2 (16.6)	54.6 (34.6)	31.9 (23.4)	65.0 (27.9)	27.1 (1.0)	58.0 (1.2)	0.038
G‐CSF	186.0 (89.6)	162.0 (50.9)	86.2 (93.7)	253.0 (60.6)	96.7 (12.2)	235.3 (1.8)	NS
GM‐CSF	95.6 (26.3)	102.7 (38.2)	59.5 (45.5)	115.4 (63.8)	50.0 (11.6)	88.6 (5.5)	0.049
IFN‐ γ	104.8 (44.5)	75.2 (34.4)	58.5 (41.9)	60.3 (24.0)	58.4 (2.3)	63.5 (7.2)	NS
IP‐10	386.0 (267.7)	940.6 (329.6)	283.6 (4173.8)	4831.2 (6826.0)	331.5 (31.4)	258.7 (4.7)	NS
MCP‐1	18.1 (9.2)	272.3 (88.8)	12.4 (318.5)	477.9 (443.4)	8.1 (3.5)	323.9 (14.4)	<0.001
MIP‐1α	24.8 (36.9)	5.8 (4.0)	6.8 (8.2)	5.0 (2.4)	5.6 (0.1)	4.0 (0.4)	NS
PDGF‐BB	1094.5(814.1)	138.7 (96.5)	997.6 (1019.0)	922.1 (1277.3)	222.3 (79.5)	173.9 (3.9)	NS
MIP—1b	169.3 (230.5)	113.4 (37.2)	67.4 (63.4)	157.3 (57.2)	55.1 (16.2)	92.3 (3.6)	NS
RANTES	181718.8 (54843.7)	2221.8 (1324.8)	128279.8 (86233.7)	6623.2 (7696.0)	36107.2 (902.8)	2215.2 (101.2)	<0.001
TNF‐α	92.4 (29.7)	56.8 (31.3)	58.1 (29.8)	54.2 (27.9)	44.5 (2.2)	49.4 (5.3)	NS
VEGF	46.9 (29.1)	35.9 (21.8)	28.0 (23.9).	57.5 (29.0)	12.6 (0)	18.3 (6.7)	NS

HSCT, hematopoietic stem cell transplantation; IL, interleukin; NS, not significant; SD, standard deviation.

a *P*‐value for comparison between all baseline and pre‐onset values.

### 3.4 Potential correlation between variables related to transplantation and abnormal production of RANTES in subjects with short‐term complications

We analyzed the relationship between the anomalous production of RANTES with chemotherapy before transplantation, the type of transplant conditioning, engraftment, and the incidence of early endothelial complications. RANTES levels at the upper limit of quantification (ULOQ) were found in 22 baseline plasma samples (28%), 18 (82%) of which developed short‐term complications. They had all received TBI‐based (11 subjects) or MCHT‐based (seven subjects) conditioning. Moreover, 19 of the 22 subjects with RANTES ULOQ levels (86%) had engraftment with a monocyte predominance (Table 7).

**Table 7:** Transplant‐related variables associated with vascular endothelial syndrome (VES) development

Variables	Whole cohort	No VES, number (%)	VES, number (%)	*P*‐value^a^
Number of patients (%)	79 (100)	52 (65.8)	27 (34.2)	—
Previous chemotherapy, number (%)				
Yes	64 (81.0)	38 (48.1)	26 (32.9)	*P* < 0.05
No	15 (19.0)	14 (17.7)	1 (1.3)	
Type of conditioning, number (%)				
TBI‐based	34 (43.0)	23 (29.1)	11 (13.9)	NS
MCHT‐based	45 (57.0)	31 (39.2)	14 (17.7)	
Type of engraftment, number (%)				
“Classical”	53 (67.1)	50 (63.3)	3 (3.8)	*P* < 0.0001
Monocyte‐predominant	26 (32.9)	2 (2.5)	24 (30.4)	
Number of baseline plasma samples (%)	26 (100)	8 (30.8)	18 (69.2)	
Rantes baseline value, number (%)				
Normal	4 (15.4)	4 (75.0)	0	*P* < 0.05
OOR >	22 (84.6)	4 (13.6)	18 (100)	
Defibrotide prophylaxis, number (%)				
Yes	44 (55.7)	42 (53.2)	2 (2.5)	*P* < 0.0001
No	35 (44.3)	10 (12.7)	25 (31.6)	

HSCT, hematopoietic stem cell transplantation; MCHT, myeloablative chemotherapy; OOR, out of range (pg/mL); TBI, total body irradiation; VES, vascular endothelial syndrome; NS, not significant.

a *P*‐values are calculated from two‐tailed exact Fisher tests.

Another close association was found between the prophylactic administration of defibrotide, a powerful endothelial protector, and the incidence of VES. Of 79 patients examined, 44 underwent prophylaxis with defibrotide (Table 6). In this group only two patients (4.5%, *P* < 0.0001) acquired a VES compared with 27 (34%) patients in the whole group.

## 4 DISCUSSION

The main feature of regular hematopoietic engraftment is the rapid normalization of the number of PMN cells and monocytes, followed by a slow reconstruction of the lymphocyte lineage.^13^ During engraftment, the percentage of monocytes in the peripheral blood of patients undergoing HSCT from HLA‐matched, HLA‐mismatched, or haploidentical donor was similar to the physiological percentage in healthy subjects.^21^ A previous German study in 1996 reported ten patients affected by temporary monocytosis with absolute values of >10 000 monocytes/μL in the early stage of engraftment.^14^ The authors attributed this phenomenon to the high number of monocytes in infused PBSCs and the use of G‐CSF at the hematologic nadir. In the current study, only one of 26 patients showing monocyte engraftment was stimulated with G‐CSF. Of note, we focused on the ratio of monocytes to PMN rather than the absolute count of monocytes. We showed that “monocyte predominant” engraftment, regardless of absolute monocytosis, was associated with a higher risk of complications such as VES.

Despite a manifold etiology of endothelial damage following bone marrow transplantation, endothelial inflammation is a common pathogenetic mechanism of the major complications of BMT. A lag occurs between endothelial damage and the clinically diagnosed syndrome. In this time frame an interplay takes place between the endothelium and the inflammatory cells recruited at the site of the damage, where several cytokines are being produced. MCP1/CCL2 is one of them and regulates the migration of monocytes, memory T lymphocytes, and natural killer (NK) cells. MCP1 induces endothelial expression of adhesion molecules, and tissue factor production switching the endothelium into a pro‐coagulant cell‐layer.^22^ MCP‐1 also modulates macrophage cytotoxicity by increasing the level of membrane bound FasL.^23^ The activation of monocytes is evidenced by the increase of TNF‐α along with IL‐15, a T‐ cell growth factor.

IL‐2 plays essential roles in the immune system, tolerance and immunity, primarily via its direct effects on T cells. In the thymus, where T cells mature, it prevents autoimmune diseases by promoting the differentiation of certain immature T cells into regulatory T cells, which suppress other T cells that are otherwise primed to attack normal healthy cells in the body. IL‐2 is produced by Th1 cells. Reduction of IL‐2 and increase of IL‐4 might suggest an alternative pattern of CD4 differentiation toward a Th2 phenotype; this hypothesis is enforced by the associated increase of IL‐5 and IL‐9 (cytokines produced by Th2 cells). IL‐4 stimulates B‐cells to produce antibodies, and Th2 activation against autoantigen will cause Type1 IgE‐mediated allergy and hypersensitivity, which, in turn, induces vascular hyperpermeability. Of interest, eosinophils are chemoattracted by IL‐5, eotaxin, RANTES and MCP1 which were all found increased (Table 5), suggesting that also acidophils may play a role in vascular endothelial syndromes.

IL‐1RA binds to IL‐1 receptor and prevents its activation. Its reduction observed after monocyte predominant engraftment could be detrimental in controlling the autoimmune response.

We hypothesized a potential relationship between the predominance of monocytes in the early engraftment stage and abnormal RANTES levels before conditioning in this group of patients. Although the levels of RANTES were not significantly higher in subjects with a prevalence of monocytes, almost all subjects with extremely high values (ULOQ) of RANTES presented with predominant monocyte engraftment. RANTES is a β‐chemokine with chemoattractant properties for monocytes and T‐lymphocytes, and is expressed on the endothelial surface of inflamed or damaged organs.^24^

Most chemokines are expressed by epithelial cells, monocytes and fibroblasts within a few minutes after cell damage, whereas RANTES is expressed 3‐5 days after the activation of T‐lymphocytes.^25^ This unusual kinetic profile of RANTES contributes to the duration of inflammation, allowing it to expand and be maintained over time.^26^ RANTES is involved in various pathological processes including immunological, degenerative, and chronic inflammatory diseases. It is also overexpressed in radiotherapy‐induced pulmonary fibrosis.^27^ Our case study included 22 clinical cases with ULOQ baseline RANTES levels, 19 of which reported monocyte engraftment. Eighteen of 22 patients with ULOQ RANTES levels underwent TBI‐based conditioning, which was performed at a different hospital. Their baseline samples were collected after six TBI fractions of 2 Gy, when admitted to our hospital. We hypothesized that TBI‐induced systemic damage alone was sufficient to cause the ULOQ RANTES levels. Not all 18 patients undergoing TBI with ULOQ RANTES levels had monocyte engraftment: three patients receiving prophylaxis with defibrotide reported regular engraftment, while monocyte engraftment was detected in the remaining 15 patients not undergoing prophylaxis with defibrotide. Indeed, multivariate analysis showed that treatment with defibrotide was inversely associated with the risk of monocyte engraftment.

The baseline samples of four patients with ULOQ RANTES levels and who did not undergo TBI treatment were collected before conditioning. Therefore, their cytokine expression could not have been affected by treatment connected with transplantation. Two of these patients were treated with sofosbuvir and underwent transplantation because of chronic active hepatitis C. Data in the literature suggests that RANTES levels are significantly higher in chronic active hepatitis C subjects.^28^ Two other patients were treated with blinatumomab before conditioning, because of relapsed leukemia. Blinatumomab is another powerful, well‐known inducer of cytokine release.^29^ None of these four patients underwent prophylaxis with defibrotide, and monocyte engraftment was detected in all of them. Our data show a significant decrease in RANTES levels to the normal range in 79 patients within 10 days after transplantation. This might be explained by the absence of cells competent to produce RANTES at the hematologic nadir: all patients were completely depleted of T‐cells.

Previous studies report a cytokinetic nadir occurring at the same time as the hematologic nadir for a high number of cytokines and chemokines.^9^ This event, however, does not explain the extremely low levels of IL‐2 found in the plasma samples of our 15 patients 1 or 2 days before the onset of early complications after transplant, and which cannot be measured using the adopted method (LLOQ). IL‐2 levels measured in these patients at the early stage of engraftment only 5‐7 days before the onset of complications were lower than the baseline values, but similar to those measured in all samples analyzed at the same stage. Not surprisingly, patients with extremely low IL‐2 levels develop a particularly severe form of GVHD, because this cytokine has a crucial role in immunologic tolerance. IL‐2^−/−^ mice develop lethal autoimmunity.^30^ A significant result of our study was that none of the 15 patients underwent prophylaxis with defibrotide. Defibrotide is a drug that acts directly on ECs and is used for the prevention and treatment of SOS, ischemia, atherosclerosis and thrombocytopenic purpura, as well as any pathological condition originating from endothelial damage.^31^, ^32^, ^33^, ^34^

## 5 CONCLUSION

Our study demonstrated that patients undergoing prophylaxis with defibrotide have a predominantly “classical” engraftment, with a common cytokine pattern and a significantly lower incidence of severe early transplant‐related complications. The results of our study demonstrate a close relationship between the type of engraftment and the onset of such complications. A predominantly monocyte engraftment might be an accessible and early predictive factor of post‐transplant complications. Given the high cost of defibrotide, selective prophylaxis may be considered for patients with monocyte engraftment.

This study had some limitations. It was a retrospective, single‐center study with a small cohort of patients. The analysis of cytokines was performed on a limited number of plasma samples. The number of patients who developed VES was too small for a comparative analysis between each type of VES, and even smaller to compare classical and monocyte‐predominant engraftments. In our opinion, further random studies will be necessary to confirm our data.

## CONFLICT OF INTEREST

None declared.
